# Integrating Nutrition and Physical Activity into the EXEMIG/01 Interdisciplinary Model for Chronic and High-Frequency Migraine

**DOI:** 10.3390/nu18121893

**Published:** 2026-06-11

**Authors:** Roberto Pippi, Deborah Prete, Stefano Pagano, Chiara Valenti, Simonetta Simonetti, Sandro Prati, Marco Alabiso, Giulia Settembrini, Daniela Fruttini, Paola Sarchielli

**Affiliations:** 1 Healthy Lifestyle Institute, C.U.R.I.A.Mo. (Centro Universitario di Ricerca Interdipartimentale Attività Motoria), Department of Medicine and Surgery, University of Perugia, Via G. Bambagioni, 19, 06126 Perugia, Italy; roberto.pippi@unipg.it; 2 Faculty of Dentistry, Department of Medicine and Surgery, University of Perugia, 06125 Perugia, Italy; stefano.pagano@unipg.it (S.P.);; 3 Master’s Degree Program in Sciences and Techniques of Sport and Preventive and Adapted Physical Activities, Department of Medicine and Surgery, University of Perugia, 06126 Perugia, Italy; simonetta.simonetti@unipg.it; 4 Postgraduate School of Orthodontics, University of Milan, Via Festa del Perdono 7, 20122 Milan, Italy; 5 Section of Neurology, Department of Medicine and Surgery, University of Perugia, Piazzale Gambuli, 1, 06129 Perugia, Italygiulia.settembrini@ospedale.perugia.it (G.S.); paola.sarchielli@unipg.it (P.S.); 6 Section of Endocrinology and Metabolism, Department of Medicine and Surgery, University of Perugia, 06129 Perugia, Italy; daniela.fruttini@unipg.it

**Keywords:** migraines, inflammation, nutrition, mediterranean diet, physical activity, interdisciplinary model, prevention

## Abstract

**Background:** Migraine (MIG) management guidelines support a comprehensive approach combining medication, therapeutic patient education (TPE), behavioral strategies, lifestyle changes, diet, and physical activity (PA). **Objective:** To present an innovative interdisciplinary outpatient model for individuals with MIG, focusing on PA, sedentary behavior, eating habits (EH), metabolic health, temporomandibular disorders, and postural dysfunctions. **Design**: A randomized controlled trial will enroll 200 adults with MIG over two years. Inclusion criteria are chronic MIG (≥15 attacks/month for ≥3 months) or high-frequency episodic MIG (8–14 attacks/month), physical inactivity, and independent walking ability. Exclusion criteria include contraindications to PA and lack of informed consent. Participants will be randomized to standard care (SC) or an intervention group receiving TPE plus three months of supervised exercise (EXE). All participants will receive an informational brochure with nutritional tips (included in SC) and undergo: (1) neurological examination, (2) validated questionnaires, (3) kinesiological and postural assessment, and (4) gnathological evaluation. The primary outcome is change in monthly MIG frequency at 6 and 12 months; additional outcomes include disability, quality of life, and intensity of MIG, PA levels, sedentary behavior, medication use, EH, functional capabilities, postural parameters, and temporomandibular disorder-related variables. **Results**: Hypothetically, the intervention may reduce monthly MIG frequency by approximately 15–20% relative to baseline. Improvements may also occur in disability, quality of life, medication use, lifestyle behaviors, and psychological and cardiometabolic parameters. **Conclusions**: This trial will evaluate whether adding supervised EXE and TPE to SC may improve MIG outcomes compared with SC alone, supporting a comprehensive management strategy.

## 1. Introduction

Migraine (MIG) is a common and disabling neurological disorder characterized by recurrent headaches and a variety of associated symptoms. Neurological disorders are the leading cause of disability globally, affecting 3.4 billion people [1], with migraines being a significant contributor. The prevalence of migraines increased from 732.56 million in 1990 to 1.16 billion in 2021. Particularly, in the 15 to 39 age group, cases rose by 39.52%, from 425.6 million to 593.8 million [2]. This trend is expected to continue, especially among males and adolescents [3,4], and the linked economic impact affects individuals, families, employers, and the broader economy [5]. Its complex pathophysiology involves genetic predispositions, neurovascular dysregulation, and environmental and lifestyle factors, all of which are crucial for informing effective treatment strategies [6,7]. The American Headache Society (AHS) [8], like other headache societies [9,10,11], supports a multidisciplinary approach for the prevention and treatment of MIG. Some authors include acute and preventive pharmacological treatment, therapeutic patient education (TPE), evidence-based behavioral interventions, lifestyle modification [12,13,14], and weight loss strategies [15,16].

In this context, the effects of eating habits (EH) and EXE on MIG management have been studied in several clinical trials [17,18,19]. These studies have demonstrated that dietary patterns may influence MIG through biological mechanisms [20], including modulation of systemic inflammation, oxidative stress, mitochondrial function, and neurotransmitter synthesis [21]. In addition, metabolic conditions that are often associated with an unhealthy diet, such as being overweight or obese and having insulin resistance, have also been linked to an increased MIG frequency and chronicity [22]. The use of standardized tools to assess overall diet quality, such as the diet risk score (DRS), may improve the identification of unhealthy dietary patterns associated with MIG and related metabolic dysfunction [23]. Nutritional factors, such as glycemic load, the intake of ultra-processed foods, and the consumption of bioactive nutrients (like omega-3 fatty acids), may contribute to the onset of MIG [24]. Research suggests that following the Mediterranean diet, rich in anti-inflammatory foods like fruits, vegetables, whole grains, legumes, and olive oil, may lower headache frequency. This effect is likely due to its ability to modulate inflammation and oxidative stress [25,26]. However, the available evidence is not fully consistent, with some studies reporting modest or variable effects. Furthermore, the causal role of specific dietary triggers remains debated. Alongside nutrition, PA has been extensively studied as a non-pharmacological strategy for MIG management; however, no direct comparison of the effectiveness of different EXE interventions has been conducted. Specifically, resistance or strength training appears to offer the greatest benefits in reducing MIG frequency [27]. This effect is likely due to targeted muscle strengthening, particularly in the core muscles of the neck, shoulders, and upper limbs [27]. The observed therapeutic effects may stem from local metabolic and neuromuscular adaptations, along with increased neck strength from resistance EXEs [28]. Regarding aerobic EXE, high-intensity aerobic workouts seem to produce superior effects compared to moderate-intensity workouts. This difference may be linked to the intensity-specific recruitment of endogenous molecules involved in EXE-related pain relief [29]. The dose-dependent impact of aerobic EXE on MIG could be mediated by improved mitochondrial and cardiorespiratory function [30] and the anti-inflammatory mechanisms associated with aerobic activity [31]. Despite these findings, the evidence remains heterogeneous, with variability in EXE type, intensity, and outcome measures, limiting the possibility of definitive conclusions.

Other relevant factors include sleep disturbances (commonly reported in individuals with MIG [32]), psychological comorbidities, temporomandibular disorders (TMDs), and postural dysfunctions, all of which may interact with lifestyle behaviors and contribute to MIG burden. Furthermore, there is increasing evidence of a significant association between temporomandibular disorders (TMDs) and primary headache [33]. People with headaches may exhibit postural alterations not only in the craniocervical region but also throughout the spine. TMDs involve various conditions affecting the masticatory muscles, temporomandibular joints, and related anatomical structures. Although several studies suggest that dietary patterns and PA may influence the frequency and severity of MIG, the current evidence remains inconsistent. Differences in study design, intervention protocols, and outcome measures limit the comparability of the findings, with some studies reporting modest or inconsistent effects.

Overall, the strength of evidence supporting integrated multidisciplinary approaches is still limited, as most studies focus on single interventions rather than combined models [34,35,36,37]. Despite the multidisciplinary and patient-centered approach to headache management in primary care showing significant benefits on outcomes and quality of life, the literature is not unanimous regarding the number and the typology of healthcare disciplines and professionals to involve, particularly those linked to nonpharmacological treatments [38]. In fact, research in this area is limited, and few studies adopt a structured interdisciplinary approach: some studies include neurologists, psychologists, and physiotherapists, while other studies also involve dentists or nurses (please see Table 1). For these reasons, there is a need for validated interdisciplinary models designed to enhance structured lifestyle-related assessment and interventions, with a specific focus on PA for individuals suffering from chronic, episodic, and high-frequency MIG.

This manuscript describes the model of the project “*Study of the effects of a multidisciplinary intervention-including physical exercise-in people with high-frequency episodic migraine and chronic migraine*”, or EXEMIG/01, an innovative model for the prevention and treatment of chronic or episodic and high-frequency MIG, based on the AHS principle of an interdisciplinary team approach. Different from previous studies, the EXEMIG/01 model integrates Migraines and Headaches Center standard care, adding an interdisciplinary structured team involving different specialists’ competence in support of the neurologist, adapting the validated C.U.R.IAMo. (Centro Universitario Ricerca Interdipartimentale Attività Motoria of the Department of Medicine and Surgery of Perugia University) work methodology [51,52]. In fact, in this model, we include EXE, nutritional assessment, postural, and gnathological evaluation. This model aims to study multiple determinants of MIG in a tailored and structured clinical manner.

## 2. Materials and Methods

**Figure 1:**
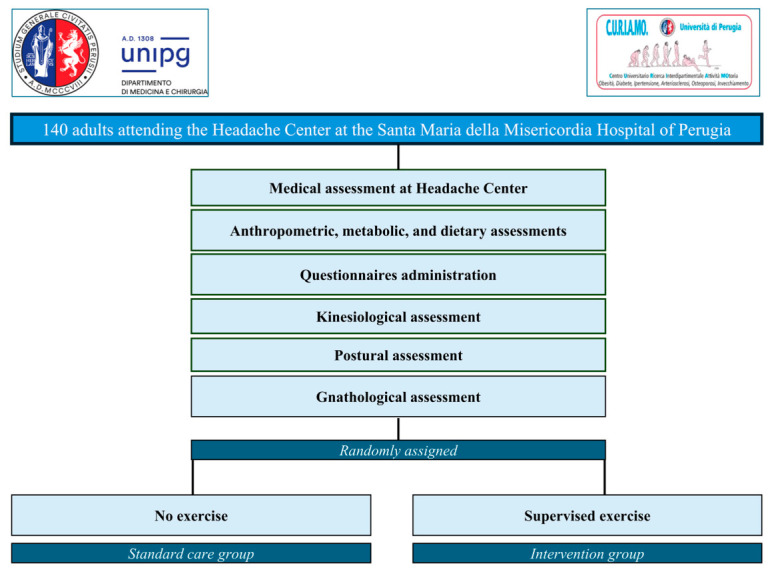
The EXEMIG01 interdisciplinary model.

The EXEMIG/01 study is a randomized, controlled interventional trial designed to present an interdisciplinary model (Figure 1) to prevent and manage MIG.

The primary outcome of the study is the change in monthly MIG frequency (number of MIG/month) from baseline at 6 and 12 months. The secondary outcomes include: MIG-related disability, MIG-specific quality of life (MSQoL), MIG intensity, PA levels and sedentary behavior, medication use (number of doses/month), dietary adherence, functional capabilities, postural parameters, and temporomandibular disorder-related variables. Exploratory measures include sleep quality, anthropometric measurements, metabolic parameters, cardiometabolic risk, psychological and psychopathological factors associated with MIG. Ultimately, the project aims to improve the treatment and management of people with MIG by providing educational material addressing known health risk factors, such as unhealthy EH (through an informational brochure given to all participants), a sedentary lifestyle, and physical inactivity.

The EXEMIG/01 trial is registered in the Clinical Trials Registry (ClinicalTrials.gov) with the identifier NCT07508449, and it was approved on 18 September 2024 by the local Ethic Committee (Comitato Etico Regionale dell’Umbria) with the protocol number CET N.4807/24.

### 2.1. Study Design

The EXEMIG/01 study was a rigorously designed, randomized, controlled, non-blinded interventional trial. This study is non-blinded due to the nature of the intervention, which may introduce performance and assessment bias, particularly with regard to self-reported outcomes. To minimize this risk, standardized procedures, validated questionnaires, and objective measures (e.g., wearable accelerometers) will be used wherever possible.

A total of 200 individuals suffering from episodic high-frequency or chronic MIG (diagnosed following the international criteria outlined in the ICHD Classification 2018 [53]) will be invited to participate voluntarily in this project. Participants will be selected from those attending the Headache Center at Santa Maria della Misericordia Hospital in Perugia, following the usual CUP booking procedures for a neurological consultation. The inclusion criteria were as follows: chronic (≥15 attacks/month, for at least 3 months) or episodic and high frequency (8–14 attacks/month) MIG; age range 18–65 years; PA levels lower than WHO recommendations (engage in 150 to 300 min of weekly moderate-intensity aerobic PA, or 75 to 150 min of vigorous-intensity aerobic activity, or a combination of both that is equivalent [54]); and ability to walk independently. The exclusion criteria were as follows: age younger than 18 years or older than 65 years; clinical evidence of serious cardiovascular, central nervous system, or significant musculoskeletal disease that may limit or contraindicate EXE; and failure to provide written consent for participation in the study.

### 2.2. Project Phases

#### 2.2.1. Phase 1: Enrollment and Medical Assessment

All participants will be invited to attend their first specialist visit to the Headache Center of the Santa Maria della Misericordia Hospital in Perugia. The neurologist conducts a thorough interview covering the onset and evolution of the headache, its frequency, duration, and intensity, associated symptoms (nausea, vomiting, photophobia, phonophobia), presence of aura, triggering and relieving factors, family history, sleep quality, stress levels, and menstrual cycle in women.

During the neurological assessment, we will collect comprehensive information for each participant, including age, sex, age of headache onset (in years), potential onset of MIG chronicity (in years), existing comorbidities, other medications being utilized, and symptomatic medications used as needed (with a focus on their efficacy). Furthermore, we will gather details regarding prophylactic medications (usage timing, dosage, potential side effects, and effectiveness: yes/no assessed as a reduction of at least 50% of attacks after at least 3 months of treatment compared to the pre-treatment period) along with the characteristics of the headaches (presence of aura, occurrence of other primary headaches, and frequency of headaches experienced in the past three months). Moreover, blood test results and any previous neuroimaging are reviewed to exclude secondary causes of headache and to assess comorbidities relevant to treatment decisions.

Based on all collected data, the neurologist formulates a diagnosis following the International Classification of Headache Disorders (ICHD-III, 2018) [53], distinguishing between migraine (with or without aura), chronic migraine (≥15 headache days/month for >3 months), tension-type headache, medication overuse headache, and other primary or secondary headache disorders. The neurologist also formulates a personalized treatment plan including acute medications, preventive pharmacological therapy (e.g., beta-blockers, antiepileptics, calcitonin gene-related peptide (CGRP) monoclonal antibodies), and non-pharmacological strategies (lifestyle modifications, biofeedback, physical therapy referral). During this phase, participants will receive detailed information about the project, and an informed consent form, along with a data processing form, will be presented for their signature.

#### 2.2.2. Phase 2: Integrated Evaluation

In addition to the medical assessment previously described, the interdisciplinary model, as shown in Figure 1, included five other steps of evaluation: (a) anthropometric, metabolic, and dietary assessment; (b) self-administered (online) and researcher-administered questionnaires; (c) assessment by a kinesiologist; (d) postural assessment; and (e) comprehensive gnathological assessment. The steps are described below.

(a) Anthropometric measurements, body composition analysis, and blood pressure assessments will be conducted. The results of any relevant blood tests, including inflammation-linked variables (such as C-reactive protein, or CRP, glycemic and lipid profile), will be documented.

**Figure 2:**
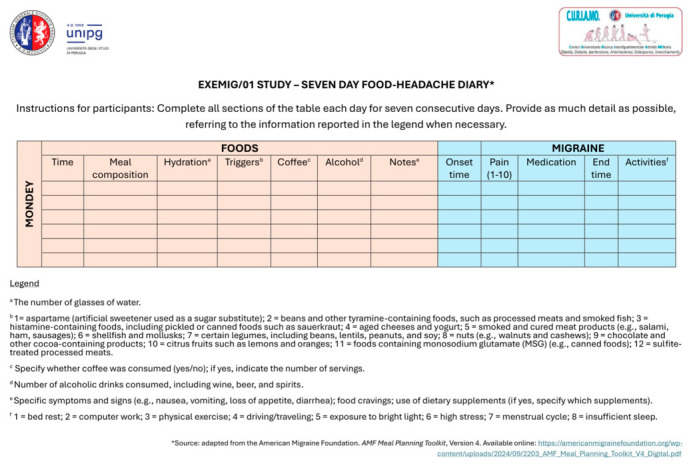
Seven day food-headache diary.

Participants will not receive specific nutritional interventions and will be encouraged to adhere to their typical EH. However, they will be instructed to meticulously track food triggers and migraine attacks by keeping a food-headache diary [55] (Figure 2).

In addition, all participants will receive a brochure with nutritional tips about EH and MIG based on guidelines and scientific literature (summarized in Table 2), including recommendations on adherence to the Mediterranean diet (rich in anti-inflammatory compounds), water, alcohol, and caffeine consumption. Importantly, these nutritional tips are provided equally to all participants as part of SC and are not part of the differential intervention between groups.

The use of a diary will enable MIG sufferers to identify and avoid individual trigger factors and could support the adoption of sustainable and health-promoting EH [56]. Overall diet quality and diet-related cardiometabolic risk using the Diet Risk Score (DRS), as well as [23] adherence to the principles of the Mediterranean diet, will be assessed.

(b) Validated questionnaires will be administered to evaluate:

- Headache progression, disabilities, allodynia, fear of movement related to headaches, and overall quality of life.
- Sleep quality, duration, and circadian rhythms.
- Presence of psychopathological disorders.
- Levels of PA and sedentary behavior among participants, related to the last seven days.
- Gnathological aspects pertinent to AXIS 1 and AXIS 2.

All the questionnaires used are presented in the *Measures* section. Participants will complete the questionnaires via a digital form divided into four parts using the Microsoft Forms platform. They will receive a link to voluntarily engage with the forms (the original versions of the project can be accessed at the following links: Part I, https://forms.office.com/e/PW6gAqWVqv (accessed on 3 June 2026); Part II, https://forms.office.com/e/t2hmcpz0Ts (accessed on 3 June 2026); Part III, https://forms.office.com/e/ghJkDLyEEj (accessed on 3 June 2026); and Part IV, https://forms.office.com/e/h6TBgmzM8a (accessed on 3 June 2026). Supervision and assistance from the study team members will be available throughout the process.

(c) Kinesiological assessment of functional capacity, including aerobic capacity, muscular strength, and flexibility, will be performed at the C.U.R.I.A.Mo. Participants will be supervised by kinesiologists specifically trained. Assessment sessions will be preceded by almost one training session to familiarize participants with the machines and the specific protocol of the test. The procedures were reported in the Measures section. Moreover, to objectively evaluate the PA habits, participants will be asked to wear an ActiGraph wGT3X-BT (ActiGraph LLC, Pensacola, FL, USA) for a week.

(d) Comprehensive postural evaluation, categorized into objective, instrumental, functional, and neuromotor examinations.

Initially, a static assessment of overall posture will be performed in an upright position on various planes, observing alignment of the head and neck, symmetry of shoulders and scapulae, spinal alignment and overhang, pelvic position, waist triangles, lower limb alignment, and foot support. The physical examination will be followed by an instrumental evaluation using a plumb line, Bunnel’s scoliometer, measuring tape, goniometer, podoscope, measuring rod, and sit-and-reach test. Barré’s vertical line will be used to determine ascending, descending, mixed, or disharmonious syndrome [57]. The examination will continue in various positions to assess localized or peripheral pain. Functional examinations will evaluate muscle elasticity, mobility, and trophic tone through anterior, lateral flexion, and global extension tests.

Trophic tones will be assessed with isotonic and isometric evaluations of abdominal muscles, gluteus medius, minimus, maximus, and spinal extensors. Evaluation of spinal curve accentuations, inversions, or extensions, especially dorsal and cervical, will be conducted in the sagittal plane to identify alterations in head posture that may affect MIGs, including specific cervical. The postural examination will conclude with neuromotor tests: Fukuda and Romberg tests (simple, monopodalic, and sensitized) will be used to assess general proprioception and balance [57].

This assessment will be followed by specific tests to evaluate how muscle contracture or weakness, cervical stiffness or instability, and poor posture may influence chronic or episodic MIG, focusing on the neck and cervical-dorsal muscles.

(e) Gnathological assessment will be conducted in accordance with the international diagnostic criteria (DC)/TMD [58]. This involves a thorough medical history of the disorders, followed by an examination of two reference axes. AXIS I focuses on identifying isolated muscle pain, condylar-disk dislocations, arthralgia, or degenerative bone changes within the temporomandibular joints, whereas AXIS II explores the psychological factors that influence the progression of the pathology and chronic pain. Participants will undergo a 4-channel surface electromyography (Teethan) examination [59]. The Baiobit motion sensor will also be used to perform cervical ROM [60,61], balance, and gait tests to identify ascending or descending interference factors in TMD.

After the evaluation, participants will be randomly assigned to two groups: the control group (*n* = 100), comprising people with MIG in standard care; and the intervention group (*n* = 100), comprising people with MIG in standard care plus supervised EXE intervention. Only the second group will attend a supervised, mixed EXE intervention (phase 3).

#### 2.2.3. Phase 3: EXE Intervention

The EXE program was set to take place at the C.U.R.I.A.Mo. of the Department of Medicine and Surgery at Perugia University. This comprehensive 12-week program will consist of two sessions each week, thoughtfully structured to include mixed activities (aerobic and muscle-strengthening EXEs), and supervised by kinesiologists graduate in Preventive and Adapted Motor Activity Sciences and Techniques (AMPA). Each session will be approximately 60 min in length and will consist of three distinct phases: warm-up, central activity, and cool-down. During the aerobic activities, participants will engage in various EXEs using ergometers, such as treadmills and cycle ergometers, designed to elevate their heart rates to a moderate training intensity, specifically targeting between 40 and 59% of their heart rate reserve. This training section plays an anti-inflammatory role, aiming to enhance cardiovascular fitness, promoting improved endurance, and overall health of the individual. In the muscle-strengthening phase, participants will focus on body-weight EXEs or with small equipment, such as elastic bands and medicine balls, as well as isotonic machines (i.e., lat machine and leg press), targeting, i.e., the neck, shoulders, and upper back. The intensity of these EXEs will be tailored to correspond to 45–50% of the individual’s one-repetition maximum (1RM), ensuring that each participant is trained while maintaining safety and effectiveness.

As participants progress through the program, and if their clinical conditions and abilities allow, there will be a carefully monitored and gradual increase in training intensity of approximately 3–5% every three weeks. The initial sessions were designed to help participants adapt to the EXEs and equipment.

The guidelines provided above serve as a framework that will be adapted to accommodate the specific EXE capacities and needs of each participant, ensuring a tailored and supportive experience for all involved. Adherence to the intervention will be monitored through attendance at supervised sessions and participation in the program. Participants’ adherence to PA recommendations will also be assessed using data from wearable devices. The EXE protocol is standardized to ensure reproducibility across sessions and participants.

#### 2.2.4. Phase 4: The Follow-Up

All evaluations will be re-executed after six (T1) and twelve (T2) months from the start of the study. During follow-up appointments, we will update specific process variables, described below.

### 2.3. Measures

#### 2.3.1. Phase 1: Medical Assessment

Participants will be invited to complete an online questionnaire to collect information about age, sex, years of illness, presence (or not) of MIG aura, frequency of headache attacks (number of attacks per month), intensity of the MIG episodes (measured using a 10-point numerical rating scale), and number of headache attacks per month (tracked using diaries provided to the participant). Moreover, dose and type of symptomatic and prophylaxis drugs used monthly by participants will be recorded.

#### 2.3.2. Phase 2: Anthropometric, Metabolic, and Dietary Assessment

The following data will be collected:

- Anthropometric and blood pressure measurements. Weight, body mass index (BMI), fat mass, lean mass, and basal metabolism will be assessed using an impedance platform (Tanita Body Composition Analyzer BC-420MA; Tokyo, Japan) or an air plethysmograph (BOD POD^®^ Composition System; COSMED Srl, Albano Laziale, Rome, Italy). Height and waist circumference will be measured using a portable stadiometer (Seca 213, Seca GmbH & Co. KG, Hamburg, Germany) and an automatically retractable measuring tape (Seca 201, Seca GmbH & Co. KG, Hamburg, Germany), respectively. Systolic and diastolic blood pressures will be recorded using a digital column sphygmomanometer designed for professional auscultatory blood pressure measurement (UM-101, A&D Medical, Tokyo, Japan).
- Hematobiochemical values. We will evaluate several parameters, including glycemia, glycated hemoglobin (also referred to as glycosylated hemoglobin or HbA1c), lipid profile (which encompasses total cholesterol, HDL, LDL, and triglycerides), CRP, and medium- to long-term coronary heart disease (CHD) risk scores.
- Dietary information. Participants will be given a seven-day food and headache diary (adapted from the American Migraine Foundation model [55]), reported in Figure 2, in order to provide a detailed and structured overview of EH and their potential relationship with MIG episodes. Participants will report their usual meal composition and timing, hydration, caffeine and alcohol intake, specific notes such as symptoms and signs (e.g., nausea, vomiting, and taking supplements), and potential food triggers, in addition to the MIG attack’s characteristics (onset, intensity, duration, and medication use). Attention will be given to identify potential dietary triggers such as alcohol, caffeine, processed meats, and food containing additives like glutamate. EH that may contribute to MIG onset or exacerbation will also be considered, such as prolonged fasting, irregular mealtimes, and inadequate hydration. In line with the latest review [62], participants will receive a brochure with nutritional tips focused on the point described above, as part of SC.

Moreover, adherence to a healthy dietary pattern will be assessed using the 14-Item Mediterranean Diet Assessment Tool (Italian version, with binary “Yes/No” responses for each item), which evaluates intake of key components such as fruit, vegetables, legumes, whole grains, olive oil, and fish [63].

In addition, overall diet quality and cardiometabolic risk will be evaluated using a Diet Risk Score (DRS), based on the consumption of key food groups associated with chronic disease risk. This is a brief 9-item diet assessment validated tool for assessing dietary patterns in clinical settings, based on the Healthy Eating Index (HEI)-2015. Excessive sodium intake and consumption of sugar-sweetened beverages, as well as insufficient intake of fruits, nuts, vegetables, and marine omega-3 fatty acids, will be considered key contributors to increased cardiometabolic risk [23].

#### 2.3.3. Phase 3: Self-Administered (Online) and Researcher-Administered Questionnaire Values

- Headache progression, disabilities, allodynia, fear of movement related to headaches, and overall quality of life. Headache-related disability will be evaluated using the Migraine Disability Assessment Score (MIDAS) [64], and the Headache Impact Test-6 (HIT-6) scores [65]. The HIT-6 is a 6-item questionnaire used to assess the impact and severity of MIG.

The MSQoL questionnaire, version 2.1, validated in Italian by Raggi et al. [66], includes 14 items assessing the impact of MIG on daily functioning across three domains: role function-restrictive (RR), role function-preventive (RP), and emotional function (EF). Items are rated on a 6-point scale (“none of the time” to “all of the time”); raw scores are summed and transformed to a 0–100 scale, with higher scores indicating better quality of life.

Allodynia will be assessed using the Allodynia Symptom Checklist (ASC), a 12-item questionnaire developed by Lipton et al. (2008) [67], which evaluates the frequency of various allodynia symptoms associated with MIG attacks.

Pain-related fear of movement will be measured using the Tampa Scale of Kinesiophobia [68].

The participants’ perspective on changes in their pain condition will be evaluated using the Patient Global Impression of Change (PGIC) [69].

Health-related quality of life will be assessed using the SF-36 questionnaire [70], while overall well-being will be measured with the World Health Organization-Five Well-Being Index (WHO-5) [71].

- Measurement of Sleep Quality, Sleep Time, and Circadian Rhythms.

Sleep onset, continuity, duration, sleepiness, and sleep-disordered breathing will be evaluated using the Sleep Scale from the Medical Outcomes Study (MOS) questionnaire [72].

Sleep quality will be assessed using the Insomnia Severity Index (ISI), developed by Bastien et al. in 2001 [73]. This is a self-assessment tool that evaluates sleep quality, the presence of insomnia, and its severity. It consists of five multiple-choice questions, with higher scores indicating a greater severity of insomnia.

Chronotype will be defined using the Morningness–Eveningness Questionnaire (MEQ-SA) created by Horne and Ostberg (1976) [74]. This self-assessment scale helps identify the participant’s circadian phenotype and consists of 19 multiple-choice questions. Higher scores indicate a morning chronotype, whereas lower scores indicate an evening chronotype.

Sleep duration will be measured using portable accelerometers, such as ActiGraph wGT3X-BT.

- Psychopathological measures. The following evaluation focuses on identifying psychopathological disorders, particularly anxious depressive symptoms, obsessive–compulsive disorder, and bipolar disorder.

The Symptom Checklist-90-Revised (SCL-90-R) [75] is a 90-item self-report instrument designed to assess psychological symptomatology in clinical and non-clinical populations. It evaluates nine primary symptom dimensions: somatization (SOM), obsessive-compulsiveness, interpersonal sensitivity, depression, anxiety, hostility, phobic anxiety, paranoid ideation, and psychoticism. In addition, the SCL-90-R includes seven supplementary items that assess appetite and sleep disorders. The instrument provides three global indices: the Global Severity Index (GSI), which reflects overall psychological distress; the Positive Symptom Total (PST), indicating the number of reported symptoms; and the Positive Symptom Distress Index, which offers insights into an individual’s response style. The SCL-90-R assesses symptom severity over the past week, capturing both internalizing and externalizing symptoms and allowing for longitudinal monitoring of psychological status.

The Beck Depression Inventory, Version II (BDI-II) (Beck, 1996) [76], is a self-assessment tool designed to gauge the severity of depressive symptoms. It is used for both clinical and research purposes and comprises 21 multiple-choice questions. Higher scores indicate more severe depressive symptoms.

The Hamilton Rating Scale for Depression, or HRSD (Hamilton, 1960) [77], is another self-administered scale that assesses the severity of depressive symptoms, serving both clinical and research needs. It consists of 21 multiple-choice questions and evaluates various domains, particularly anxiety/somatization, weight changes, cognitive disorders, diurnal variations in symptoms, psychomotor slowing, and sleep disturbance. Higher scores reflect greater severity of depressive symptoms.

The Hamilton Rating Scale for Anxiety (Hamilton, 1959) [78] is a self-administered rating scale designed to measure the severity of anxiety symptoms for both clinical and research purposes. It includes 14 items that are completed by the clinician based on participants’ observations during the interview. Higher scores indicate more severe anxiety symptoms.

The Spielberger State-Trait Anxiety Inventory (STAI-Y) (Spielberger et al., 1983) [79] is a self-administered assessment that measures the level of anxiety symptoms. This scale consists of 40 multiple-choice questions that distinguish between state anxiety and trait anxiety.

The Mania Rating Scale (MRS) (Young, 1978) [80] is a self-administered tool that evaluates hypomanic and manic symptoms and their severity. It comprises 11 multiple-choice questions, where a higher score indicates increased symptom severity.

The Mood Disorder Questionnaire (MDQ) (Hirschfeld, 2002) [81] is a self-administered scale that assesses the likelihood of an individual being affected by bipolar disorder. It consists of 17 multiple-choice questions.

The Obsessive-Compulsive Inventory-Revised (OCI-R) (Foa et al., 2002) [82] is a self-administered rating scale that identifies and assesses the severity of obsessive–compulsive disorder. This scale contains 18 multiple-choice questions, with higher scores reflecting greater severity of obsessive–compulsive disorder.

- Levels of PA and sedentary behavior. The International Physical Activity Questionnaire (IPAQ) short form [83] assesses PA levels (expressed in MET-hours/week) by studying time spent in different types/intensity PA subgroups (moderate, vigorous, walking) and sedentary activities (<1.5 METs) over the past 7 days [83]. It is a self-report questionnaire whose responses are used to calculate metabolic equivalent task (MET) minutes per week, which allows classification of participants into low, moderate, or high PA levels.
- Gnathological aspects pertinent to AXIS 1 and AXIS 2. Gnathological assessment will be conducted according to the DC/TMD framework, evaluating both Axis I (physical and clinical features) and Axis II (psychosocial and behavioral factors).

1. Axis I: general information, pain, and symptoms will be observed through the Demographic Questionnaire, the TMD Pain Screener [84], and the Symptom Questionnaire DC/TMD [84,85].
2. ASSE II: pain intensity and disability will be assessed using the GCPS Chronic Pain Scale (CGPS 2.0 [86]); changes in functional limitation will be assessed using the Jaw Functional Limitation Scale (JFLS-8) [87]; severity of depression will be measured using the Patient Health Questionnaire (PHQ-4) [88]; and the frequency and quantification of jaw overuse behaviors will be assessed with the Oral Behavior List (OBC) [89].

#### 2.3.4. Phase 4: Assessment by a Kinesiologist

The participant’s levels of PA and sedentary behavior will be objectively measured using portable accelerometers, specifically the ActiGraph wGT3X-BT [90]. These devices, which are worn on the wrist, continuously record data movements, which are processed to calculate time spent in sedentary, light, moderate, and vigorous activity. People will be invited to wear the ActiGraph wGT3X-BT for a week and to record lifestyle habits (PA, EXE, sedentary time) in a specific diary. Aerobic capacity will be evaluated through the 6-Minute Walking Test [91]. Dynamic muscle strength will be assessed by estimating the one-repetition maximum (1-RM) through submaximal strength tests of the upper and lower limbs. These tests will be performed using natural weights or isotonic machines, applying the Brzycki 1-RM prediction equation [92]. Isometric hand grip strength will be measured with a DynX^®^ dynamometer (Akern Srl, Pontassieve, Florence, Italy), according to standard procedures [93].

Additionally, we will detect perceived effort and training intensity using the Borg Rating of Perceived Exertion (RPE) scale (6–20) [94] along with continuous heart rate monitoring via a heart rate monitor.

#### 2.3.5. Phase 5: Postural Assessment

Cervical flexion-extension assessments are conducted in sitting, prone, or supine positions. Beyond evaluating mobility, these assessments also examine the subject’s capacity to control movement [95].

In the head rotation test, the participant rotates their head to the right and left while the examiner stabilizes their shoulders, in a seated or standing position. In addition to assessing mobility, the examiner can evaluate the tension of the trapezius muscle, both when people maintain a forward gaze during head rotation. The participant may also be queried regarding what enters their field of vision in relation to the examiner, such as the hand, elbow, shoulder, ear, eye, or nose.

The lateral neck flexion test is employed to evaluate asymmetries or limitations in cervical lateral flexion and to explore potential associations with pain or MIG symptoms. During this assessment, the participant is seated and instructed to laterally flex their heads. The range of motion (ROM) is measured using a goniometer, and the onset of cervical pain is recorded on a visual analog scale (VAS) ranging from 0 to 10. Additionally, the presence of pressure or MIG symptoms, such as nausea, dizziness, or radiating pain, is noted. An asymmetrical ROM between the right and left sides may indicate a mechanical cervical dysfunction, which is frequently associated with MIG [95].

The neck flexor strength and endurance test evaluates muscle endurance deficiencies, muscle pain, and potential headaches associated with impaired isolated muscle endurance of the deep neck flexors. During the assessment, the participant lies supine on a bed and elevates their head approximately 2.5 cm while maintaining a tucked chin position. The examiner records the duration for which the subject sustains this posture; a duration exceeding 30 s in women and 38–40 s in men is deemed normal [96].

The Cervical Extensor Strength Test is designed to evaluate the isometric strength of the neck extensor muscles, specifically the upper trapezius, splenius, and semispinalis. During this assessment, the participant lies prone on a bed, allowing the head to extend beyond the edge, with the chin slightly retracted. The individual is required to maintain the head in a neutral position against gravitational force, or with slight manual resistance, until failure occurs. The duration for which the participant can sustain this position is recorded. A time span ranging from 60 to 90 s, contingent upon age and gender, is deemed normal. Premature failure or any compensatory movement in the lumbar region suggests diminished endurance of the cervical extensors and compensatory hyperactivation [96,97].

In the test for the strength and endurance of the middle trapezius and lower trapezius, the subject lies prone on the bed, arm abducted at 90°, elbow extended, and thumb pointing upwards; they lift their arm, trying to keep the scapula adducted with the arm raised against gravity. From here, resistance is applied to the forearm to stabilize the other scapula [97].

In the test for the strength and endurance of the lower trapezius muscle, the participant lies prone on the examination table with the upper limb abducted at 120° and thumb pointing upward; the upper limb is slowly raised while keeping the scapula depressed and abducted [97].

#### 2.3.6. Phase 6: Comprehensive Gnathological Assessment

A comprehensive gnathological assessment will be performed to obtain a multidimensional profile of the participant’s craniofacial and mandibular health, integrating both clinical and behavioral dimensions. This process begins with a structured clinical questionnaire designed to collect detailed information on medical history, previous traumas, dental and medical treatments, systemic conditions, and current symptoms. It further investigates habits and parafunctional behaviors, including bruxism, nail-biting, gum chewing, tongue pressure, and the use of visual orthopedic devices. Additionally, sleep quality, occupational posture, and potential associations between stress and symptom onset are explored. Physical features, pain, and functional limitations will be evaluated through Axis I, while psychosocial factors, emotional status, and behavioral patterns will be studied using Axis II.

Muscular function during static and masticatory activities will be studied with a 4-channel surface electromyographic examination (Teethan) [59].

Ascending or descending interference factors in temporomandibular disorders will be identified through the cervical ROM tests, balance tests, and walking tests, using the Baiobit [60,61].

#### 2.3.7. Follow-Up Assessment

During this phase, we provided some process variables, as follows, to evaluate the feasibility, fidelity, and overall quality of the study’s execution.

Protocol Adherence. The extent to which participants and investigators followed the study’s predefined procedures, including eligibility, intervention delivery, and assessment schedules.

Completion of Questionnaires. The percentage of participants who fully completed all self-reported outcome measures at each designated time point in the study.

Patient Participation. The engagement level of participants in study activities, including attendance, assessments, and cooperation.

Dropouts. The number of participants who left the study early, with reasons such as personal choice, adverse events, loss to follow-up, or investigator decision.

Adverse Events. Any unintended harmful occurrences experienced by participants during the study were evaluated for frequency, severity, and connection to the study intervention.

Adherence to Study Timelines. The degree to which recruitment, intervention delivery, and data collection adhered to planned schedules.

We also intend to conduct telephone outreach to participants who choose to withdraw from the study.

### 2.4. Statistical Analysis

The primary efficacy analysis will be performed in the intention-to-treat (ITT) population. The pre-protocol population consisted of all patients in the ITT population without major protocol deviations and will be used for the supportive analysis of the primary efficacy endpoint.

The quantitative data collected, organized into two distinct groups of subjects, will be presented in tables that include the mean and standard deviation. In addition, qualitative data will be represented through frequency distributions.

The primary endpoint (number of MIGs/month from baseline to 6 and 12 months) will be analyzed using a linear mixed-effect model (LMEM) with treatment group × time as the fixed effect and the baseline number of MIG/month value as the covariate.

For qualitative data analysis, the Chi-square test will be implemented.

The scores obtained from the evaluation scales will be correlated with the clinical parameters of the participants, when applicable, using the Pearson or Spearman correlation coefficients, contingent upon the distribution of the data.

In the case of individuals with MIG, a logistic regression analysis will be performed to investigate potential associations between psychopathological parameters and the response to headache treatment. Adherence to both PA and TPE will be incorporated as process variables in the analyses. Dietary adherence scores from the Mediterranean Diet Assessment Tool, Diet Risk Score, and food-headache diaries will be assessed longitudinally for their potential as predictors of migraine outcomes. Additionally, EXE adherence will be evaluated through attendance records and wearable device data.

Analyses will be limited to participants with complete data on different measurements. We hypothesized that missing data occur randomly into two group. We plan to gather additional information from participants who drop out.

Should the dropout rate exceed our projections, we will employ a regression model to impute the missing data.

The exploratory outcomes will be evaluated utilizing the same LMEM employed for the primary analysis, without adjustments for multiple comparisons. Conclusions will be derived from effect size estimates, specifically Cohen’s d, accompanied by a 95% bootstrap confidence interval (CI). Effect sizes will be systematically classified as small (d = 0.2), medium (d = 0.5), and large (d = 0.8).

All statistical analyses will be completed using IBM SPSS^®^ Statistics for Windows version 25.0 (IBM Corp., Armonk, NY, USA).

*Sample Size.* The sample size calculation was based on real-life data regarding the response rate to therapy through PA in people with MIG, specifically focusing on the number of headache days. According to the literature, this approach indicates a decrease of approximately 15–20% relative to baseline data [98]. To determine the number of participants in the intervention and control groups, we referenced various articles on the subject, particularly the work of Krøll et al. [98,99], which indicates that the average difference in the number of headache days is almost 2 days less than the baseline. The standard deviation is estimated to be approximately 50% of the average for both groups. Assuming a 1:1 ratio, with a significance level of 5% and a statistical power of 80%, a sample size of a total of around 200 participants (100 patients per group) will be determined, accounting for an anticipated dropout rate of 30%. The recruitment of this cohort will be scheduled for over 9 months to effectively achieve the desired enrollment.

## 3. Expected Results

As this is a study protocol, no actual results are currently available. The following section outlines the study’s hypotheses and anticipated outcomes based on existing evidence. According to the current literature, the proposed interdisciplinary intervention, primarily based on supervised physical EXE and TPE, is hypothesized to improve clinical outcomes in individuals with MIG (e.g., frequency) and may also contribute to improvements in lifestyle-related outcomes and overall disease burden [100].

Regarding the primary outcome, the study aims to evaluate whether the intervention is associated with a reduction in MIG frequency. It is hypothesized that the intervention group may show a decrease in monthly headache days of approximately 15–20% compared with baseline [98]. This assumption is in line with previous evidence, including studies by Krøll et al. [98,99], which reported an average reduction of about two headache days per month in people engaging in PA interventions.

Regarding the secondary outcomes, in accordance with previous studies [14,17,62], we hypothesize that the intervention may be associated with improvements in MIG-related disability, MSQoL, MIG intensity, PA levels, and sedentary behavior, medication use (number of doses/month), dietary adherence, functional capacity, and postural and temporomandibular function, in line with the intervention’s interdisciplinary nature [101]. With respect to explorative measures, potential improvements may be observed in sleep quality [102], weight and WC [103,104], and key cardiometabolic parameters (including fasting glucose, HbA1c, lipid LDL, HDL, triglycerides, blood pressure, and CRP) and related risk, which may reflect changes in systemic inflammation and cardiometabolic risk, particularly among participants in the supervised EXE program [105,106]. However, these outcomes will be explored and require confirmation.

Additional benefits are anticipated in other areas, including anxiety and depression symptoms, and kinesiophobia. A reduction in allodynia scores is also hypothesized, suggesting a potential modulation of central sensitization mechanisms [107].

Although no structured nutritional intervention is planned, all participants will receive nutritional tips on EH and MIG based on guidelines to counteract the inflammatory and metabolic background of MIG, as suggested by recent literature [108]. We hypothesize that, over time, participants may show small improvements in dietary awareness, adherence to Mediterranean diet principles, and overall diet quality following the nutritional tips provided during phase 2 of the study. Particularly, participants may show greater adherence to the Mediterranean diet, higher overall diet quality (as assessed by the DRS [34]), and healthier eating behaviors, such as more regular mealtimes, improved hydration, and reduced intake of ultra-processed foods. Participants are expected to develop greater awareness of food potentially linked to an attack, although no definitive evidence supports a direct causal relationship. The use of a diary may help identify individual dietary trigger factors.

## 4. Discussion

MIG is a complex neurological disorder characterized by multifactorial etiology involving genetic, neurobiological, and environmental components [109]. Its effective management generally requires a comprehensive approach targeting modifiable risk factors to homeostasis and overall psychophysical well-being. In this context, an integrated model combining pharmacological treatment with lifestyle TPE and interventions may help reduce the burden of acute attacks and may also contribute to enhancing participants’ self-efficacy and long-term disease management, with potential benefits for quality of life. To the best of our knowledge, in recent years, relatively few studies (Table 1) have adopted a multidisciplinary approach to support individuals with MIG, and none have presented a similar, integrated model. In this context, our manuscript proposes an interdisciplinary, structured model that combines professionals’ profiles, such as neurologist, nutritionist, kinesiologist, and gnathologist profiles, in MIG management. While multidisciplinary approaches have previously been suggested, the present model intends to combine a multimodal assessment framework with these components within a single, clinically applicable model.

Given that MIG often is a social stigma for many people [110,111,112], it is imperative to focus on the role of lifestyle factors [12] and associated comorbid conditions that can exacerbate headache symptoms [112]. Specifically, according to other authors, considerations related to PA [36], nutritional aspects [113], postural alignment [114], gnathological issues [33], and psychopathological factors [115] represent potentially relevant targets to be addressed within a comprehensive management strategy. This comprehensive approach may support MIG management and contribute to a broader understanding of the multifactorial nature of headache disorders.

There is growing evidence to suggest that increased cardiometabolic risk, driven by factors such as systemic inflammation, dyslipidemia, impaired glucose metabolism, and poor diet quality, may contribute to the onset and severity of MIG. This highlights a shared pathophysiological link between metabolic health and MIG disorders from a nutritional perspective.

Elimination diets have been proposed as a strategy to identify and remove potential dietary triggers of MIG attacks; however, their overall efficacy remains modest. Although numerous foods have been implicated, convincing evidence currently supports a trigger role only for alcohol and caffeine [116]. Nonetheless, studies by Alpay et al. have explored elimination protocols based on foods associated with elevated IgG antibody levels (e.g., spices, seeds, nuts, seafood, vegetables, cheeses, fruits, and sugary products), reporting reductions in attack frequency, mean attack number, and pain intensity [37,117,118]. Nutritional strategies may benefit from being tailored to the individual’s pathophysiological profile and subsequently optimized through targeted nutrient supplementation. Certain nutrients may help prevent or ameliorate MIG by modulating oxidative stress. Given the significant variability in how individuals respond to dietary factors, keeping a food diary may help identify which foods trigger or alleviate MIG episodes [108,119]. In this context, using structured food and headache diaries and validated questionnaires could help to improve monitoring of EH and adherence to nutritional recommendations over time. Although the study does not include a standardized dietary intervention, the longitudinal collection of dietary data may help to identify behavioral patterns potentially associated with MIG frequency and severity.

According to recent studies, regular personalized PA [36] has been associated with a reduction in systemic inflammation (maybe linked to modulation of endorphin and serotonin systems), and improvement in sleep and mood. Moreover, scientific evidence shows that physical inactivity [120], poor EH [113], being overweight, and mood or sleep disorders contribute to the chronicity of the disease. Compared to previous experiences [121], this model may contribute to the ongoing development of interdisciplinary strategies for MIG management, incorporating conventional pharmacological therapies with a structured regimen of TPE and supervised EXE, while also including the assessment of EH as a non-differential component across groups [109]. Despite being relatively underexplored in the literature, some TPE experiences are starting to be documented [122,123], but to our knowledge, none of these include nutritional, neurological, posturological, gnathological, and kinesiological assessment plus supervised PA. This aspect is mainly innovative because EXE is considered a trigger by people with MIG, and they tend to avoid it [124], developing kinesiofobia [125]. Moreover, MIG pain limited PA in people who suffered [124]. The proposed model may help guide individuals with MIG toward engaging in PAs within a supervised and individualized setting, customizing the program according to postural and gnathological assessment. In fact, certain temporomandibular disorders and postural alterations can aggravate symptoms, promoting muscle tension and the activation of trigeminal nociceptive circuits [33]. Improving these aspects may contribute to reducing peripheral nociceptive inputs, preventing recurrent activation of the trigeminal-vascular system. Thanks to improved neuromuscular function and neurogenic inflammation, a reduction in allodynia and central sensitization can be expected [67].

For the previous considerations, an interdisciplinary approach, involving professionals such as neurologists, dieticians, kinesiologists, and gnathologists, allows for a more comprehensive assessment and may act on multiple aspects relevant to MIG management. This approach could promote greater treatment adherence, reducing dependence on medication and improving quality of life, laying the foundation for stable and lasting change in the lifestyle of individuals with MIG. In this context, TPE serves as a crucial resource for enhancing people’s awareness. It encourages individuals to recognize their specific triggers and fosters the development of self-management skills [126,127]. Our protocol is designed to evaluate whether a reduction of approximately 15–20% in monthly attacks and improvements in clinical metrics can be achieved. The goal is not only to achieve immediate clinical benefits but also to encourage long-lasting lifestyle changes among participants.

Additionally, our study could contribute to the development of a potentially replicable interdisciplinary model that can be applied in headache centers and in general medicine and rehabilitation settings. It also aims to promote the implementation of the EXE recommendations. However, there are potential limitations to consider.

A limitation of this study is its unblinded design, as it is impossible in behavioral intervention research like EXE. Both participants and evaluators knew group assignments, which can introduce bias and potentially overestimate the intervention’s effectiveness. Participants in the intervention group, aware of receiving active treatment and engaging regularly with a kinesiologist, may have developed heightened expectations of improvement, potentially amplifying perceived reductions in pain and quality-of-life gains beyond the actual physiological effects of EXE (enhanced placebo effect). Conversely, control group participants, conscious of not receiving active intervention, may have experienced demoralization, contributing to reduced diary compliance, higher dropout rates, or an overestimation of symptom burden (e.g., demoralization). Furthermore, given the inherently subjective nature of pain assessment, intervention group participants may have been inclined to report more favorable outcomes to justify their investment in the treatment, introducing a degree of social desirability or reporting bias. Finally, the difference between the intervention and control groups likely stems from both the actual impact of exercise and psychological effects due to the lack of blinding, which may result in a larger reported effect size than in blind studies. Moreover, it is crucial to underline that our model will be initially applied to one center (the Headache Center of Perugia). This single-headache center recruitment may limit the study’s generalizability, as participants were drawn from a single clinical site, potentially introducing selection bias. This could affect the external validity of the results, as individuals at one center may not represent the broader migraine population. Future multicenter studies with diverse participants are needed to confirm and extend these findings.

Participant adherence to both the EXE program and TPE (including nutritional tips) may vary and could potentially influence the study outcomes. To address this aspect, adherence to PA will be monitored through attendance records and wearable-device data, while EH will be observed using food-headache diaries and validated dietary tools, including the 14-item questionnaire and the DRS. These measures may help clarify the relationship between lifestyle behaviors and MIG-related outcomes. As this is a study protocol and diary data are not currently available, the diary structure and selected variables have been designed to identify behavioral patterns and potential temporal associations between EH and MIG outcomes. Recently, some authors have suggested that remote management of PA could provide benefits in this context [119,127]. The requirement for specialized personnel adds complexity to the implementation of the protocol, highlighting both its challenges and benefits, as noted by Pradelli et al. [128].

Future studies should examine how long the observed benefits last over time, as well as the effectiveness of digital or telematic interventions in supporting follow-up care.

## 5. Conclusions

In the context of MIG, a complex and multifactorial “functional disorder”, a multidimensional approach that extends beyond pharmacological treatment and considers individual lifestyle factors, including EH and metabolic health, may be relevant for clinical management. This study aims to investigate whether an integrated and personalized interdisciplinary approach, characterized by collaboration across various professional disciplines, may contribute to reducing MIG frequency and improving patient-reported outcomes, including quality of life. Within this framework, PA and TPE are the core active components of the intervention; nutritional aspects are included as additional components that act across all groups, potentially modulating metabolic and inflammatory pathways and promoting sustainable lifestyle behaviors. In conclusion, a further strength of the proposed model is the simultaneous assessment of PA behaviors, sedentary time, and EH using both subjective and objective tools. The synergistic interplay among medical treatment, physical education, psychology, and nutrition represents a promising and potentially sustainable framework for the management of chronic MIG, thereby possibly contributing to a broader perspective in the care of this intricate condition.

## Figures and Tables

**Table 1:** Multidisciplinary studies.

Authors (Year)	Study Type	Specialists Involved
Golovacheva et al. (2025) [39]	Randomized Controlled Trial (RCT)	Neurologists, CBT specialists, and therapeutic gymnastics specialists.
Sujan et al. (2025) [40]	RCT	Yoga instructors (in addition to standard neurological care).
Tahzeeb et al. (2025) [41]	Cross-sectional Study	Recommends a team including Neurologists and Dentists/Gnathologists for TMD comorbidity.
Hisham et al. (2023) [42]	RCT	Neurologists, researchers, and experts in relaxation training
Helmerson et al. (2021) [43]	Pilot Study	Team for a group-based “migraine school” (like nurses, physiotherapists, psychologists).
Golovacheva et al. (2021) [44]	Case Report	Neurologist, CBT specialist.
De Almeida Tolentino et al. (2021) [45]	RCT	Neurologists, Physiotherapists (including those trained in pain neuroscience education), and researchers
Evans et al. (2020) [46]	RCT	Nutrition scientists, Neurologists, Psychologists, and obesity Specialists
Aguirrezabal et al. (2019) [47]	RCT	Family doctors and neurologists
Cramer et al. (2019) [48]	Prospective Observational Study	Team delivering conventional and complementary medicine (exact composition not specified).
Gaul et al. (2011) [49]	Observational Study	Neurologists, behavioral psychologists, physiotherapists, sports therapists, and specialist headache nurses.
Magnusson et al. (2004) [50]	Comparative Study	Neurologist, psychologist, occupational therapist, and physiotherapist.

**Table 2:** Nutritional tips for patients with MIG.

Topic and Authors	Practical Recommendations for Patients with MIG
Mediterranean dietary pattern Behrouz V. et al. (2025); Di Lorenzo C. et al. (2023); Arab A. et al. (2023) [20,22,25]	Encourage adherence to a Mediterranean-style dietary pattern rich in vegetables, fruit, legumes, whole grains, nuts, extra-virgin olive oil, and fish; limit ultra-processed foods and excessive saturated fats.
Meal regularity Seng E.K. et al. (2022) [12]	Maintain regular meal timing and avoid prolonged fasting or skipping meals, especially breakfast.
Hydration Ashina M. (2021) [6]	Promote adequate daily hydration and regular fluid intake throughout the day.
Caffeine intake Seng E.K. et al. (2022); Gazerani P. (2020); Nguyen K.V. & Schytz H.W. (2024) [12,34,37]	Moderate and consistent caffeine intake is preferred; excessive consumption or abrupt withdrawal should be avoided. Patients should monitor individual sensitivity.
Alcohol consumption Poboży T. et al. (2025) [26]	Limit alcohol intake, particularly red wine and binge drinking; identify individual trigger patterns.
Ultra-processed foods Cavestro C. et al. (2025); Tu Y.H. et al. (2025) [21,56]	Reduce intake of ultra-processed foods, processed meats, and foods rich in additives when individually associated with attacks.
Trigger foods Poboży T. et al. (2025); Roldán-Ruiz A. et al. (2025); Nguyen K.V. & Schytz H.W. (2024) [26,35,37]	Encourage individualized identification of potential trigger foods using headache and food diaries rather than generalized restrictive diets.
Omega-3-rich foods Di Lorenzo C. et al. (2023); Chen, T.B. et al. (2024) [22,24]	Increase intake of omega-3-rich foods such as fatty fish, walnuts, and seeds.
Weight management Cavestro C. et al. (2025) [21]	Promote healthy body weight through sustainable dietary and physical activity interventions.
Behavioral self-management Demarquay G. et al. (2021); Hisham S. et al. (2023) [9,42]	Integrate dietary education with stress management, relaxation techniques, and therapeutic patient education.

## Data Availability

No new data were created or analyzed in this study. Data sharing is not applicable to this article.

## Abbreviations

The following abbreviations are used in this manuscript:

1-RM	One-Repetition Maximum
ASC	Allodynia Symptom Checklist
BDI-II	Beck Depression Inventory Version II
BMI	Body Mass Index
CHD	Coronary Heart Disease
DRS	Diet Risk Score
EF	Emotional Function
EH	Eating Habits
EXE	Exercise
GCPS	Graded Chronic Pain Scale
GSI	Global Severity Index
HDL	High-Density Lipoprotein
HRSD	Hamilton Rating Scale for Depression
ISI	Insomnia Severity Index
IPAQ	International Physical Activity Questionnaire
JFLS-8	Jaw Functional Limitation Scale
LDL	Low-Density Lipoprotein
MEQ-SA	Morningness–Eveningness Questionnaire
MIDAS	Migraine Disability Assessment Score
MSQoL	Migraine-Specific Quality of Life
MRS	Mania Rating Scale
MOS	Medical Outcomes Study Sleep Scale
OBC	Oral Behavior Checklist
OCI-R	Obsessive-Compulsive Inventory–Revised
PA	Physical Activity
PGIC	Patient Global Impression of Change
PHQ-4	Patient Health Questionnaire-4
PSDI	Positive Symptom Distress Index
PST	Positive Symptom Total
RPE	Borg Rating of Perceived Exertion
RP	Role Function–Preventive
RR	Role Function–Restrictive
SF-36	Short Form 36 Health Survey
STAI-Y	Spielberger State-Trait Anxiety Inventory
TG	Triglycerides
TMD	Temporomandibular Disorder
TPE	Therapeutic Patient Education
VAS	Visual Analog Scale
WHO-5	World Health Organization-Five Well-Being Index
